# Annexin A1: Uncovering the Many Talents of an Old Protein

**DOI:** 10.3390/ijms19041045

**Published:** 2018-03-31

**Authors:** Madeeha H. Sheikh, Egle Solito

**Affiliations:** The William Harvey Research Institute, Barts and the London School of Medicine and Dentistry, Queen Mary University of London, London EC1M 6BQ, UK; m.h.sheikh@qmul.ac.uk

**Keywords:** ANXA1, inflammation, immune repair, cancer, CNS, HPA axis

## Abstract

Annexin A1 (ANXA1) has long been classed as an anti-inflammatory protein due to its control over leukocyte-mediated immune responses. However, it is now recognized that ANXA1 has widespread effects beyond the immune system with implications in maintaining the homeostatic environment within the entire body due to its ability to affect cellular signalling, hormonal secretion, foetal development, the aging process and development of disease. In this review, we aim to provide a global overview of the role of ANXA1 covering aspects of peripheral and central inflammation, immune repair and endocrine control with focus on the prognostic, diagnostic and therapeutic potential of the molecule in cancer, neurodegeneration and inflammatory-based disorders.

## 1. Introduction

Annexin A1 (ANXA1), a 37-kDa protein, belongs to the annexin superfamily of calcium-dependent phospholipid-binding proteins [1]. The molecule is regulated by glucocorticoids and inhibits the action of cytosolic phospholipase A2 (PLA2), subsequently blocking the release of arachidonic acid and in turn preventing synthesis of eicosanoids (e.g., prostaglandins, thromboxanes, prostacyclins and leukotrienes) [2,3,4,5,6]. The protein mediates its pharmacological effects through binding to G-protein coupled receptor (GPCR) formyl peptide receptor 2 (FPR2), and/or by binding to the phospholipid bilayer of cell membranes [7,8,9,10]. Notably, it is not only the full-length ANXA1 molecule which has the ability to mediate its widespread effects. ANXA1’s N-terminal-derived peptides Ac2-26, Ac2-12 and Ac2-6 have also been reported to induce activation of FPR1 and FPR2 [11,12,13].

Since its discovery, ANXA1 has proven to be responsible for controlling more than the activation of PLA2, with evidence to support the protein’s role in blocking leukocyte extravasation, inducing apoptosis and modulating cytokine synthesis [14], as well as controlling hypothalamic–pituitary–adrenal (HPA) axis physiology [15] and regulating the tightness of the blood–brain barrier (BBB) [16]. In fact, the role of ANXA1 has been investigated in a variety of different fields including cardiology, neurology, endocrinology and oncology. This review aims to recapitulate the last 30 years of research surrounding ANXA1’s multiple pathophysiological roles as well as its potential to act as a therapeutic and diagnostic agent.

## 2. Annexin A1 and Inflammation

Annexin A1, formerly renocortin, macrocortin or lipocortin-1, was first identified in the 1980s by Francoise Russo Marie and Rod Flower, as a factor regulated by dexamethasone with the potential to inhibit cPLA2 [17,18]. *ANXA1* gene structure presents a non-translated first exon, a long intron and four repeats (highly similar among the annexins) suggesting a gene duplication theory [19,20].

Clear evidence of ANXA1’s role in inhibiting cPLA2 was provided through the generation of mice lacking the *ANXA1* gene, in which levels of cPLA2 mRNA and protein were elevated compared to wild-type mice [21], supporting earlier in vitro studies conducted using U937 cells [22]. 

The initial identification of ANXA1 as an anti-phospholipase protein paved the way for this molecule to be investigated as an anti-inflammatory agent. As such, ANXA1 was shown to control the release of nitric oxide, as seen in macrophages treated with dexamethasone in conditions such as sepsis or under LPS stimulation, resulting in the inhibition of inducible nitric oxide synthase (iNOS) and simultaneously up-regulating production of potent anti-inflammatory cytokine IL-10 [23]. It is widely established that IL-10 production can be increased by the ERK signalling pathway that is activated upon ANXA1 binding to its FPR thereby suggesting the presence of a looping mechanism to control both IL-10 and nitric oxide release [24]. Interestingly, ANXA1 also inhibits cyclo-oxygenase-2 (COX-2) expression, hence, controlling pro-inflammatory mediator release; a phenomenon unique to microglial cells [25].

### 2.1. Transcriptional and Translational Control of ANXA1

The role of glucocorticoids in controlling ANXA1 mRNA transcription has been observed in vitro, in vivo and ex vivo. In an in vivo model using Wistar rats treated with either Methylprednisolone or a 21-aminosteroid, there was upregulated ANXA1 expression in all areas of the brain compared to non-treated controls. Ex vivo analysis of alveolar macrophages, obtained by broncho-alveolar lavage, from patients with inflammatory lung disease (i.e., bronchial asthma) undergoing treatment with glucocorticoids also showed higher ANXA1 expression [26]. Similarly, in vitro studies conducted using a human lung epithelial cell line A549 showed upregulation of ANXA1 expression when treated with phorbol-myristate (PMA) and interleukin 6 (IL-6) which was mediated by the activation of the transcription factor NF-IL-6 (C/EBP β) [6,27,28]. Aside from transcription, IL-6 and dexamethasone cause ANXA1 translocation to the cell surface and secretion which has important implications in controlling the binding and attachment of leukocytes onto endothelial cell surfaces [29,30,31]. Co-localization studies of ANXA1 with the ABCA1 transporter in follicular stellate cells of the pituitary is another example of ANXA1 secretion, although in this specific case, it is restricted to the pituitary gland [32].

Moreover, post-translational modifications of ANXA1 are important in allowing its translocation to the cell surface for further secretion which is independent of the classical ER/Golgi exocytosis pathway [29] as it does not contain a signal sequence to enter into these sub-cellular compartments [33]. Serine-27 phosphorylation along with lipidation [30] induces ANXA1 translocation onto the membrane via MAPK, PI3K and PKC-dependent signalling cascades [31]. In contrast, the phosphorylation on tyrosine 21 mediated by EGF signalling would localize the protein at a cytoplasmic level [34]. Involvement of these signalling pathways is critical as they activate a number of secondary messengers including ERK, JNK, p38 and Akt; these are crucial in regulating many cellular processes such as stress responses, metabolism, cell cycle events and apoptosis [35,36,37]. The pathway-dependent translocation of ANXA1 to the cell surface for binding to its GPCR therefore represents a paracrine mechanism used to control, dampen and limit the immune response, underlining the potential of ANXA1 to be used as a therapeutic. 

### 2.2. Annexin A1 in the Innate Immune System

A key anti-inflammatory property of ANXA1, and perhaps the most documented, is the ability of the protein to inhibit leukocyte (neutrophil and monocyte) transmigration. Initially, this was discovered through the administration of dexamethasone to mice with zymosan peritonitis, subsequently inhibiting leukocyte accumulation; an effect reversed with immunisation against recombinant ANXA1 [38]. Using a similar model, Lim et al. showed detachment of neutrophils from the vascular endothelium wall of mesenteric post-capillary venules in mice treated intravenously with ANXA1 with a marked reduction in the number of cells migrating through the tissues [39]. In tandem, upregulated ANXA1 levels in circulating leukocytes, following glucocorticoid treatment, were reported to be responsible for prolonging diapedesis time of leukocytes compared to controls [40]. Mechanistically, the above-described effects are correlated to ANXA1’s ability to modulate adhesion molecule-based leukocyte-endothelium interactions. Studies in the monocytic U937 cell line observed ANXA1 to co-localise with α4β1 integrin on leukocytes to prevent interaction of α4β1 with vascular cell adhesion molecule (VCAM)-1 and/or to cause l-selectin shedding from leukocytes in a calcium-dependent manner thereby preventing the tethering, rolling and firm adhesion of leukocytes to the endothelium for transmigration [10,41]. Leukocytes, upon cellular activation by chemokines, mobilise their endogenous ANXA1 to the plasma membrane [42] and this secreted ANXA1 serves to promote leukocyte detachment from endothelial cells, thus serving as a negative regulator of the transmigratory process. Indeed, in human neutrophils >60% of cytoplasmic ANXA1 is stored in gelatinase granules for rapid mobilisation/secretion [43].

Another major involvement of ANXA1 in resolution of inflammation is through accelerating apoptosis. Transfection of monocytic U937 cells with full length recombinant ANXA1 constitutively activates caspase-3 activity [44]. Moreover ANXA1 stimulates an intracellular increase in cytosolic calcium resulting in the dephosphorylation of the Bcl-2-associated death promoter (Bad), thus, activating the apoptotic effector machinery [45,46]. Alongside initiating apoptosis, ANXA1 also serves as a regulator of apoptotic cell removal. Engulfment of apoptotic cells requires presentation of ligands to act as signalling molecules to phagocytes. ANXA1 is recruited to the cell surface of apoptotic cells in a caspase-and calcium-dependent manner to co-localise with ‘eat-me’ signal phosphatidylserine [47] implying that ANXA1 may serve as a safety measure to ensure dead cells are removed effectively and efficiently without causing inflammation. This concept was further supported through a study by Scannell et al., who showed that pre-conditioned medium from pro-apoptotic neutrophils released pro-phagocytic factors for promotion of removal by macrophages, in which ANXA1 and its peptide derivatives were abundant [48]. 

Crucially, the above mentioned anti-inflammatory effects can occur by both the full length ANXA1 protein or by the protein’s cleaved N-terminal peptides. Confirmation of these results is provided through investigations in *ANXA1*^−/−^ mice that exhibit enhanced migratory properties of leukocytes, upregulated l-selectin expression and abnormal phagocytosis by macrophages [21,49].

### 2.3. Annexin A1 in Adaptive Immunity

Over the years, research has shown that ANXA1 is also involved in adaptive immunity, although notably the expression of ANXA1 in T-cells is approximately 100-fold lower than in neutrophils and macrophages [50]. Addition of human recombinant ANXA1 (hrANXA1) to T-cells stimulated with CD3 and CD28 increases T-cell proliferation and activation [51] leading to increased cell surface expression of the FPR1 on T-cells in parallel with mobilisation of ANXA1. The overall combination of these events causes prolonged stimulation of AKT and ERK pathways thereby controlling T-cell proliferation through modulation of T-cell receptor signal strength [52]. In addition, the AP-1, NFAT and NF-κB pathways are also initiated [51]; the activation of all three suggests that ANXA1 has the ability to control multiple aspects of cell activation making it an even more attractive therapeutic molecule in modulating inflammatory responses [53]. *ANXA1^−/−^* T-cells lack the ability to engage these signalling pathways [54]. 

Remarkably, one particular study has suggested the potential of ANXA1 in modulating the differentiation of naïve T-cells into T-helper cells (T_H_). D’Acquisto et al., found ANXA1 to promote the development of T_H_1 cells (IFN-γ, TNF-α & IL-2 producing) and to suppress development of T_H_2 cells (IL-4, IL-5, IL-6, IL-10 & IL-13 producing) [51]. In another study, it was seen that ANXA1-derived peptide Ac2-26 inhibited the proliferation and cytokine production of both T_H_1 and T_H_2 cells by interfering with T-cell activation mechanisms dependent on antigen presentation [55]. Of note, no further studies to date have been able to show ANXA1’s involvement in T_H_-cell differentiation, in contrast ANXA1 deficiency has been shown to cause proliferation and activation of T_H_17 cells correlated with autoimmune pathology of the retina. In humans, ANXA1 deficiency can be associated with the autoimmune disease, uveitis. In a mouse model, hrANXA1 administration rescued the severity of the disease through restricting T_H_17 development and thus pro-inflammatory cytokine secretion via regulation of SOCS3/STAT3 signalling [56].

**Figure 1:**
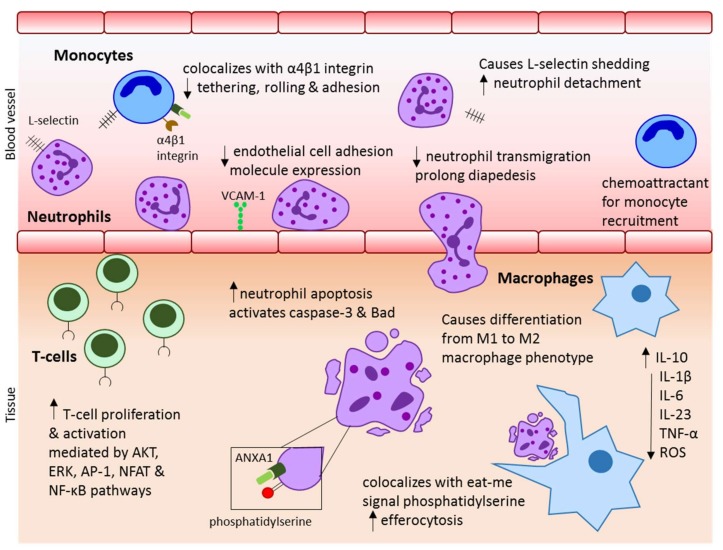
Summary of the role ANXA1 in controlling inflammation through its effect on immune cells.

Without a doubt, further research is necessary to understand the effect of ANXA1 on CD4^+^ vs CD8^+^ cells, differentiation of T-regulatory cells, Th17 cells and B-cell development. An overview of the anti-inflammatory mechanism of ANXA1 on the immune system is provided in Figure 1.

## 3. Annexin A1 and Immune Repair

Recent work has focused on the role played by ANXA1 and its peptides specifically in immune repair. Particular focus has been placed on macrophages as it was noticed, in biopsies from patients with inflammatory bowel disease (IBD), that ANXA1 localisation is higher in macrophages during disease resolution compared to active disease where instead ANXA1 expression is greater in neutrophils [57]. ANXA1 from apoptotic neutrophils acts as a chemoattractant to recruit monocytes for differentiation into macrophages for the detection, removal and presentation of foreign antigens thereby playing a key role in the resolution phase of inflammatory responses [58]. ANXA1 increases efferocytosis and absence of ANXA1 has been correlated with reduced phagocytosis of bacterial and fungal particles [59]. In Mycobacterium tuberculosis, ANXA1 is essential to provide immunity as it assists in dendritic cell antigen-presentation for CD8^+^ T-cell stimulation. Absence of ANXA1 results in an impaired CD8^+^ T-cell response alongside reduced dendritic cell-mediated efferocytosis [60]. 

Macrophages can be differentiated into either a pro-inflammatory M1 phenotype or anti-inflammatory M2 phenotype. In ischemia-reperfusion induced acute kidney injury of mice, inflammatory M1 macrophages are present immediately after injury followed by the accumulation of tissue-repair M2 macrophages; suggesting the role of these cells in resolving inflammation [61]. In presence of ANXA1, liver macrophages are induced towards a M2 phenotype expressing high IL-10 and low IL-12p35 [62] and inhibiting expression of pro-inflammatory cytokines IL-1β, IL-6, IL-23 and TNF-α. The dampening down of the pro-inflammatory macrophage phenotype has also been demonstrated ex vivo using non-alcoholic steatohepatitis livers isolated from mice treated with recombinant ANXA1, triggering suppression of M1 macrophages concurrent with an increase in IL-10 mRNA [63].

Furthermore, the stimulation of the P2X7 receptor on macrophages, independent of polarisation state, results in the release of ANXA1. In M1 macrophages, the P2X7 receptor activated the NLRP3 inflammasome, which is uncoupled on the M2 macrophages [64]. Interestingly, the expression of the receptor is higher in M2 macrophages and does not result in production of reactive oxygen species (ROS), suggesting an alternative mechanism to the pro-resolving mechanism of ANXA1 [65].

The majority of work investigating the ability of ANXA1 to participate in immune repair has been carried out in IBDs in which the epithelial barrier is disrupted resulting in chronic inflammation and mucosal wounds [66]. Severe IBDs such as Ulcerative Colitis (UC) or Crohn’s can be treated using anti-TNF-α therapies in order to control the inflammatory response. Evaluation of biopsies from patients under anti-TNF-α therapy for UC show increased ANXA1 expression in the mucosal crypts and epithelium. In contrast, patients receiving no treatment who experienced a flare of UC did not express ANXA1 in the intestinal epithelium [57]. In an animal model of colitis, induced by either dextran sodium sulphate or 2,4,5-trinitro benzene sulfonic acid and treated with ANXA1 peptide MC-12, colonic inflammation was reduced as measured by NF-κB activation, myeloperoxidase, PGE_2_, COX-2 and pro-inflammatory cytokine (TNF-α, IFN-γ, IL-1β and IL-6) levels [67].

Wound healing is an essential component of immune repair and activation of the FPR1 receptor has been shown to induce intestinal epithelial cell migration and wound closure. Stimulation of the FPR1 receptor by both ANXA1 and its peptide Ac2-26 generates superoxide species via NADPH oxidase 1 (NOX1), causing phosphorylation and activation of focal adhesion kinase (FAK) which is involved in cell migration and proliferation. Mice lacking NOX1 present with defects in intestinal wound repair following injury, which is rescued by administration of ANXA1 [68]. More recently a study by Alam et al., uncovered the ability of specific gut microbiota species to activate the FPR1 receptor and induce cell migration through ROS generation from NOX1 [69]. 

Another manner in which ANXA1 provides repair is through positive alteration of the fibrotic response. In a bleomycin-induced pulmonary fibrosis model, ANXA1 absence led to a more severe fibrosis phenotype with increased lethality that was rescued upon Ac2-26 peptide treatment [66]. Similarly, mice with silicosis present with improved lung function and pathology measured by reduced leukocyte infiltration, collagen deposition, granuloma formation and fibroblast activation when treated with ANXA1 mimetic peptide [67]. Treatment with ANXA1 could hold therapeutic potential in other fibrosis-driven diseases such as kidney disease, whereby ANXA1 in renal fibroblasts reduces α-SMA and collagen A1 gene expression due to inhibition of TGF-β induced signalling and cytokine production [68]. Likewise, ANXA1 treatment in RA patients reduces scar formation through increasing MMP-1 presence for the degradation of extracellular matrix components [69].

### Targeting ANXA1 in Extracellular Vesicles

ANXA1 is often found in the extracellular matrix and its presence can be accounted for by the release of the protein by extracellular vesicles (EVs), including larger microparticles (100–1000nm) and smaller exosomes (40–100nm) in which ANXA1 is localised [70]. The growing interest of nanoparticles as a therapeutic delivery strategy, has implicated the potential to exploit and harness ANXA1 in EVs as a method of promoting inflammation resolution.

ANXA1-containing EVs are released endogenously from intestinal epithelial cells upon raised pro-inflammatory cytokines and help to promote wound repair; this resolution ability is lost in EVs derived from *ANXA*^−/−^ mice. Delivery of nanoparticles containing ANXA1 mimetic peptide Ac2-26 in both a murine model of colitis and colonic-induced wounds, show enhanced wound healing and recovery following a single intramuscular injection [71]. Moreover, as ANXA1-contaning EVs are elevated in patients with IBD, these EVs could serve as a biomarker of inflammation. 

The prospect of ANXA1-containing nanoparticles to deliver therapeutic benefit has also been investigated in RA and atherosclerosis. As cartilage is impenetrable to the passage of cells, neutrophils secrete microvesicles containing ANXA1 to enter the cartilage. In RA patients, neutrophil-derived microvesicles overexpressing ANXA1 are increased in concentration within the synovial fluid compared to plasma. These microvesicles have the ability to prevent loss of proteoglycans in order to maintain cartilage [72]. 

Previous work by Dreschler et al., implicated the role of ANXA1 and its peptide Ac2-26 in inhibiting integrin activation and myeloid cell accumulation to arterial walls by chemokines CCL5, CCL2 and CXCL1 [73]. In *Apoe*^−/−^ mice fed a high fat-diet, repeated intravenous administration of Ac2-26 reduced atherosclerotic lesion size and lesional macrophage accumulation. Therefore, the therapeutic potential of collagen IV-targeted nanoparticles containing Ac2-26 was evaluated in a separate study using fat-fed *Ldlr*^−/−^ mice. It was seen that; the release of Ac2-26, from the nanoparticles accumulated at the vessel lesions, induced plaque stability and reduced atherogenesis [74]. Furthermore *Apoe*^−/−^ mice lacking the FPR2 receptor, showed significantly increased lesion size, pinpointing the ANXA1-FPR2 interaction as an important repair mechanism in atherogenesis [73].

## 4. Annexin A1 and Cancer

Dysregulation of ANXA1 levels and alterations to its sub-cellular localization have been associated with the development and progression of a large number of cancers.

Elevated levels of ANXA1 have been detected in lung cancer [75,76], colorectal cancer [77,78,79], hepatocellular carcinoma [80], pancreatic cancer [81] and in melanomas [82]. In these particular forms of cancers, high expression of ANXA1 is positively correlated with disease severity and increasing tumour stage. In fact, in an antithrombin III SV40 T large antigen (ASV) transgenic mouse model that constitutively develops hepatocellular carcinoma, high ANXA1 levels precede tumour development [34]. In the same manner, higher expression levels of ANXA1 were detected in sera obtained from patients with lung-cancer compared with matched high-risk controls, who did not go on to develop lung cancer [83], thus suggesting the potential for ANXA1 to act as a biomarker for cancer diagnosis and prognosis.

Additionally, up-regulated ANXA1 promoted tumour invasion and metastasis as seen in colorectal cancer whereby ANXA1 levels were raised in sentinel lymph nodes compared to normal lymph nodes [84]. Similarly, in a murine model of melanoma ANXA1 levels increased by 2.5-fold in B16B16 metastatic cells versus non-metastatic B16F10 cells. An siRNA knock-down of ANXA1 in the B16B16 cells reduced their invasive capability giving rise to idea that ANXA1 could also be a potential therapeutic target for metastatic cancers [82]. This has been analysed in hepatocellular carcinoma, colorectal carcinoma and lung cancer whereby treatment to respective cell lines of HepG2 by Sorafenib [80], HCT116 by Indomethacin [85] and A549 cells by Eurycomanone [86] resulted in significant down-regulation of ANXA1 protein expression. Further studies are required to evaluate the effectiveness of targeting ANXA1 for cancer therapies. 

Conversely, particular cancers are more prone to develop in downregulated states of ANXA1 expression including cancers such as prostate [87,88,89], cervical [90,91,92], lymphoma [93], oesophageal [90,91,92], larynx [94], nasopharyngeal [95] and oral squamous cell carcinoma [96,97]. Interestingly, in oesophageal cancer, ANXA1 levels were reportedly lower in moderately and poorly differentiated tumours in comparison to well-differentiated types and levels were downregulated particularly at the transitional switch between dysplastic to invasive tumours suggesting that ANXA1 could act as an early indicator for cancer progression [90,98]. A similar phenotype was seen in nasopharyngeal cancers in which primary tumours had lower ANXA1 expression when compared to lymph node metastasised tumours [95].

In prostate cancer, reduced ANXA1 expression has been linked to prostate acinar morphogenesis due to increased IL-6 presence [99] suggesting the importance of ANXA1 in mediating cytokine expression. Re-introduction of ANXA1 into prostate cancer cells induces a pro-apoptotic effect through blocking EGF-mediated proliferation and activation of p38 and JNK pathways [100]. Other studies in prostate cancer have also found a link between histone deacetylase inhibition, ANXA1 and apoptosis [101]. 

The multiple effects induced by ANXA1 serve to indicate its potential as a pharmacological agent, however, further studies are required to understand the exact mechanisms of ANXA1-mediated cellular activation. This is particularly important in cancers such as breast, bladder, gastric and leukaemia, whereby it has been discovered that ANXA1 can behave as both a tumour suppressor and oncogene (reviewed in [102,103]), with differentiated levels of expression dependent on tissue and cell type, emphasising the need for personalised medicine in the future. 

### Localization of ANXA1 and Its Effect on Tumorigenesis

ANXA1 is known to have three distinct sub-cellular locations; the cytoplasm (ANXA1 contributes to 2%–4% of cytoplasmic protein), the nucleus and the plasma membrane [104]. Annexins do not contain a nuclear targeting sequence, nonetheless its presence in the nucleus may be related to certain conditions. For example, investigations into oesophageal squamous cell carcinoma have shown ANXA1 to be highly expressed on the nuclear membrane, which is absent in normal oesophageal epithelial cells. Moreover the nuclear expression patterns of ANXA1 are significantly associated with pathological type, histological grade and prognosis [105,106]. ANXA1 nuclear translocation has also been negatively associated with the survival of patients with oral squamous cell carcinoma [107] and gastric adenocarcinoma [108]. The significance of ANXA1 in the nucleus is its ability to exert helicase activity, once sumoylated, leading to DNA replication [109]. ANXA1 can then be mono-ubiquinated and in the presence of carcinogenic heavy metals such as AS^3+^ and Cr^6+^, in place of Ca^2+^, trans-lesion synthesis and mutagenesis occur leading to tumourigenesis [110]. It is thought that the initial translocation of ANXA1 into the nucleus, however, requires mitogenic, proliferative and DNA-damaging stimuli such as EGF, heat, hydrogen peroxide or phorbol 12-myristate 13-acetate [111,112]; all known processes to induce oncogenesis. 

## 5. Annexin A1 in the Endocrine System

The HPA axis is a major neuro-endocrine organ which regulates many body processes including immune response, mood, digestion, energy storage/expenditure and stress. Glucocorticoids are important in regulating the magnitude and duration of these responses, specifically by controlling the release and gene expression of corticotrophin (ACTH) and corticotrophin-releasing hormone/arginine vasopressin (CRH/AVP) [113]. Not only does ANXA1 mimic the glucocorticoid-induced secretion of these hormones [114] but use of neutralizing anti-ANXA1 sera or antisense oligodeoxynucleotides (ODNs) directed specifically against ANXA1 reverses the glucocorticoid-mediated effects [114,115]; revealing an ANXA1-dependent mechanism of HPA axis regulator. In fact ANXA1 also controls the actions of glucocorticoids on other pituitary hormones including prolactin [116], luteinising hormone [117] and thyrotropin (TSH), particularly in response to pro-inflammatory cytokines [118]. 

Gonadal function relies on the levels of glucocorticoids; in prolonged levels of stress the HPA axis is altered leading to disruption in glucocorticoid production and release which can therefore affect fertility. ANXA1 mimics the inhibitory effect of glucocorticoids on testosterone secretion, and may be a contributor to stress-induced infertility [119]. However, this same mechanism of steroid inhibition can be beneficial during pregnancy and lactation whereby ANXA1 and oestrogen interaction leads to reduced stress and incidence of autoimmune inflammatory diseases by lowering levels of ACTH and CRH secretion [120,121,122]. In fact female *ANXA1*^−/−^ mice examined during pregnancy show altered oestrogen cycles, exacerbated inflammatory reaction and enhanced plasma progesterone at the start of the pregnancy resulting in fewer births [123] implicating the importance of ANXA1 interaction with endogenous steroid production and regulation. Certainly, ANXA1 expression is increased in the placenta at term of normal pregnancies [121] whereas lower levels of ANXA1 are present in the placentas from Gestational Diabetes Mellitus pregnancies that have a high level of inflammatory cytokines [124]. In fact a number of studies report an association of oestrogen-mediated protective effects through ANXA1 pathway activation in lymphocytes in both systemic and cerebral inflammation [125,126,127,128,129]. 

Early studies by Melki et al. found ANXA1 to attenuate the insulin response through inhibiting the activity of the insulin receptor kinase [130]; notably the insulin receptor was purified from a human placenta. On the other hand studies conducted in rat pancreatic cells and MIN6N8a cells (an insulin secreting cell line) found ANXA1 to increase insulin secretion through cell-surface binding [131,132]. Furthermore, insulin secretion was synchronous with phosphorylated ANXA1 upon exposure to high glucose levels and results found ANXA1 to be localized within the insulin-containing vesicles suggesting an autocrine-mechanism of action [133]. Evidence suggests that ANXA1 expression may vary in different tissues and thus may account for contrasting results. However, the importance of ANXA1 to be a regulator of insulin action should not be dismissed, as this could have far-stretching effects on the role of the protein in conditions of obesity and metabolic syndrome where insulin resistance is a key contributor to disease progression. Insulin receptor engagement activates the Akt pathway, which plays a central role in regulating cellular metabolism [134]. Indeed, ANXA1 attenuates microvascular complications (nephropathy and cardiomyopathy) through restoration of Akt signalling in a murine model of Type I Diabetes Mellitus (T1DM) [135].

## 6. Annexin A1 in the Central Nervous System 

In the last couple of decades, research into ANXA1 has implicated a strong and important role of the protein in the brain, where it provides neuroprotective and repair functions. ANXA1 is expressed in a number of cells of the neurovascular unit including brain endothelial cells, microglial cells, astrocytes, pericytes and neurons.

### 6.1. Blood–Brain Barrier (BBB)

The BBB is a physiologically active barrier that is present in the brain microvasculature to protect the central nervous system (CNS) against damage from invasion of toxins, pathogens and peripheral inflammation. It is composed of brain endothelial cells (linked by tight junctions and adherens junctions), pericytes, astrocytes and the basal lamina; to give rise to an effective barrier with low paracellular permeability to the movement of molecules, ions and immune cells [136,137]. ANXA1 has a key involvement in maintaining BBB integrity, achieved through co-localisation with actin microfilaments present at the tight junctions between cells. ANXA1 is seen to be highly concentrated at the site of cell-cell contact with *ANXA1*^−/−^ mice exhibiting loss of tight and adherens junction proteins occludin and VE-cadherin respectively [16,138]. The disruption to junctional proteins and actin microfilaments results in the loss of cell polarity, which has important implications for the localization and distribution of transporters present on brain endothelial cells. This has downstream effects on the supply of essential nutrients and ions to meet the metabolic demand of the brain. 

Importantly, the brain endothelial cells express the FPR2 receptor and it has been demonstrated by Cristante and colleagues that ANXA1 signalling inhibits the activity of small GTPase RhoA. RhoA itself causes the destabilization of the actin cytoskeleton, further implicating ANXA1 involvement in BBB maintenance. In fact, *ANXA1^−/−^* mice showed constitutively elevated BBB permeability compared to wild-type mice as measured by MRI, trans-endothelial transport of Evans blue and extravasation of serum IgG [16]. Loss of BBB permeability has been proposed as an effect of aging. Since ANXA1 is an essential component of the BBB tightness and it expression declines with aging in fibroblasts [139] [139] and leukocytes [140] we may speculate that ANXA1 downregulation at the BBB is responsible for BBB leaking. This has important implications for the development and progression of age-related neurodegenerative disorders such as Alzheimer’s disease, as well as accounting for overall susceptibility to infections with increasing age. 

Of note, ANXA1 presence can be traced to pre-natal brain development [121], with expression of ANXA1 detected in the BBB endothelial cells and microglial-like cells during foetal development [138]. Moreover the protein is involved in the morphogenesis of organs including the kidney, lung, salivary glands, placenta and inner ear [121] suggesting that ANXA1 mediates downstream effects that are crucial for maintaining body homeostasis in all stages of life. 

In Multiple Sclerosis, the loss of ANXA1 expression has been identified at brain parenchymal capillaries of patients [16]. The loss of expression occurred at sites distant from the active lesion suggesting that the disruption of BBB is required for enhanced leukocyte extravasation leading to the damage of myelin sheaths seen on neurons. A vital role of ANXA1 is in limiting leukocyte extravasation through preventing α4β1 integrin-VCAM1 interaction. In fact, one of the key treatments for Multiple Sclerosis—Natalizumab is a humanized monoclonal antibody against α4β1 integrin with the aim to prevent T-cell infiltration into the brain parenchyma thus suggesting the potential for exploiting ANXA1 as a therapeutic particularly as Natalizumab carries the risk of developing progressive multifocal leukoencephalopathy (PML) as a side effect [141]. PML is a rare viral disease of the brain, normally kept under control by the immune system however a dampened immune system in conditions such as MS allow for the viral disease to be activated resulting in fatal inflammation. As ANXA1 is an endogenous protein it is likely to have a lower potency of inducing severe immunosuppression, however this remains to be investigated. Moreover, ANXA1 deficiency has been linked to the severity of other autoimmune diseases including Uveitis [56] and T1DM [135] and interestingly hrANXA1 treatment can reduce the severity of such autoimmune diseases. It would be interesting to understand if there is a particular immune cell signature that underlies the development and progression of these diseases.

ANXA1 may also confer a therapeutic potential in protecting against stroke, as seen using a murine-model of stroke induced by mid-cerebral artery occlusion. Treatment with ANXA1 Ac2-26 peptide provided cerebro-protection, via FPR2, by reducing infarct volume, leukocyte adherence and markers of inflammation whilst improving neurological scores compared to non-treated mice [142]. A similar effect was also seen when using an alternative ANXA1 analogue 1-188 [143].

### 6.2. Annexin A1 and Brain Immunity

Microglia are the brain tissue-resident macrophages sensing infectious and metabolic disturbances and consequently aid in maintaining brain homeostasis. In events of neurological disease or brain injury, microglial cells are activated [144,145]. In the peripheral system, macrophages are involved in phagocytosis and ANXA1 is known to encourage the removal of apoptotic cells in order to resolve inflammation. In a similar manner, it was first shown that microglia-derived ANXA1 is required for the efficient removal of apoptotic neurons by co-localising with the phosphatidylserine present on the cell surface to serve as a phagocytosing signal to microglia via the FPR2 receptor in a autocrine-paracrine fashion [146]. Critically, microglia become constitutively activated under inflammatory conditions for example in Alzheimer’s disease in response to Aβ, and we have shown that ANXA1 treatment reduces both in vitro and in vivo the levels of Aβ by increasing its enzymatic degradation by neprelysin and to stimulate Aβ phagocytosis by microglia [147].Pharmacological intervention through exogenous ANXA1 treatment restored microglial surveillance to ensure resolution and maintenance of brain homeostasis; implicating the use of ANXA1 in neurodegenerative disorders [146,147]. A similar effect of ANXA1 peptide Ac2-26 was seen in ischemia-injury of neurons, whereby the peptide-FPR engagement promoted the transition of activated microglia from an inflammatory M1 phenotype to a resolving M2 phenotype as well as inducing the migration of these microglial cells [148]. These studies implicate that ANXA1 treatment in the brain provides neuroprotection. The upregulation of ANXA1 in microglia has also been detected in models of transmissible spongiform encephalopathies and excitotoxic neuronal injury, specifically in the sites surrounding the lesions [149,150,151], suggesting a compensatory mechanism responsible for the immune repair. 

### 6.3. Peripheral Inflammation, Neuroinflammation and the Potential Role of ANXA1

As the BBB is the major site of communication between the peripheral system and the brain, there is evidence to suggest that increased peripheral inflammation in conditions such as Type II Diabetes Mellitus (T2DM) have the ability to cause endothelial dysfunction at the level of the brain microvasculature, leading to neuro-inflammatory disorders and cognitive decline.

Rodent models of obesity and diabetes show reduced numbers of pericytes and activation and infiltration of microglia [152,153] with high-fat feeding (approximately 45% fat) leading to neuronal losses [154,155], suggesting BBB disruption and induction of a local inflammatory response. Flow cytometric analysis in diabetic mice revealed infiltration of macrophages into the perivascular space and activation of microglial cells [155]. Post-mortem studies of patients with diabetes reveal reduced grey and white matter in the hippocampus region [156,157,158] suggesting a connection between metabolic disorders and brain deficit. Dementias are characterised by progressive memory loss and cognitive decline, corresponding to the loss of neurons and brain matter in the hippocampus of diabetic patients [159,160]. Aside from dementias, there is also strong evidence to suggest that T2DM increases the risk and severity of stroke and cerebral ischemic [161,162,163,164]. 

Circulating levels of ANXA1 have been reported to be downregulated in T2DM [165] although it is important to note that the cohort of this study was very limited and no patient clinical information were reported. Given that ANXA1 is involved in tight junction formation and inhibiting leukocyte adherence, it is highly plausible that ANXA1 can provide protection against such inflammatory disorders. Our preliminary data suggest that high-fat fed mice treated with ANXA1 show improved BBB tightness, reduced leukocyte migration and improved metabolic activity of brain endothelial cells further implicating the many aspects by which ANXA1 can provide improvement in heath state. 

## 7. Conclusions

**Figure 2:**
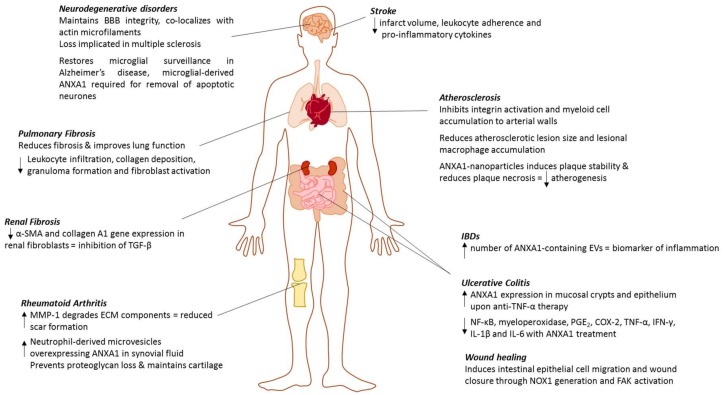
Summary of the involvement and therapeutic potential of ANXA1 in various disease states within the body.

ANXA1 has widespread effects across the body, ranging from early development through to immune regulation and disease development, in this review we emphasize its potential as a therapeutic target and/or biomarker in a number of diseases (Figure 2), further suggesting that its role to date has yet to be fully unveiled. A vast amount of research, both non-translational and translational, is further required to understand the exact implications of altering the expression levels of the protein in a spatio-temporal manner; however, it is without doubt that ANXA1 functions as far more than just as an anti-phospholipase protein.

## Abbreviations

ANXA1	Annexin A1
PLA2	cytosolic phospholipase A2
GPCR	G-protein coupled receptor
FPR2	hormyl peptide receptor 2
HPA axis	hypothalamic–pituitary–adrenal axis
BBB	blood-brain barrier
iNOS	inducible nitric oxide synthase
VCAM-1	vascular cell adhesion molecule -1
Bad	Bcl-2-associated death promoter
hrANXA1	human recombinant Annexin A1
RA	Rheumatoid Arthritis
IBD	Inflammatory Bowel Disease
UC	Ulcerative Colitis
NOX1	NADPH oxidase 1
FAK	focal adhesion kinase
EVs	extracellular vesicles
ACTH	corticotrophin
CRH	corticotrophin-releasing hormone
AVP	arginine vasopressin
PML	progressive multifocal leukoencephalopathy
T2DM	Type II Diabetes Mellitus
